# Preoperative Immunonutritional Indices in Colorectal Cancer: The Contribution of Albumin, Time-Dependence of Effect, and Threshold Transportability in a Saudi Cohort

**DOI:** 10.3390/curroncol33090496

**Published:** 2026-08-22

**Authors:** Moaz W. Abulfaraj, Ali H. M. Farsi

**Affiliations:** Department of Surgery, Faculty of Medicine, King Abdulaziz University, Jeddah 21589, Saudi Arabia; afarsi@kau.edu.sa

**Keywords:** colorectal cancer, prognostic nutritional index, serum albumin, systemic inflammation, emergency surgery, disease-free survival, cut-off transportability, Saudi Arabia

## Abstract

Preoperative immunonutritional indices are widely reported to predict survival after colorectal cancer (CRC) resection, yet their independence varies across cohorts and no data exist from the Arab Gulf. In this retrospective cohort study we analyzed 316 patients undergoing curative resection for stage I–III colorectal adenocarcinoma at a Saudi tertiary center between 2013 and 2022, of whom 48 (15.2%) presented as emergencies. The prognostic nutritional index (PNI) and a composite albumin–neutrophil-to-lymphocyte ratio (albumin–NLR) score were assessed against overall survival (OS) and disease-free survival (DFS) using Cox models adjusted for age, sex, emergency presentation, tumor site, neoadjuvant therapy, adjuvant chemotherapy and lymphovascular invasion and stratified by stage and American Society of Anesthesiologists class. Over a median follow-up of 58.1 months there were 88 deaths and 124 DFS events. The PNI independently predicted OS (adjusted hazard ratio 0.958, 95% CI 0.929–0.987) and DFS (0.964, 0.940–0.988); the albumin–NLR score did not. Albumin alone carried the signal (OS 0.938), with lymphocytes, neutrophils and the NLR all null. The PNI effect was confined to the first 36 months (0.944 versus 1.003 thereafter), and published cut-offs classified 70.8% of the cohort as high-risk. Immunonutritional prognostication in CRC is albumin-driven, time-limited and sensitive to cut-off provenance.

## Simple Summary

Colorectal cancer is one of the most common cancers, and surgery offers the best chance of cure. Doctors would like a simple way, using blood tests already taken before surgery, to identify patients more likely to fare badly so that extra support can be arranged. Scores combining albumin, a protein measured routinely in blood, with white blood cell counts have been proposed, but findings differ between studies and none have been tested in patients from the Gulf region. We studied 316 patients who had surgery for colorectal cancer in Saudi Arabia. Albumin, rather than the white blood cell counts, explained the link with survival; the link applied only to the first three years after surgery; and threshold values borrowed from other countries did not fit our patients. Locally derived thresholds are needed.

## 1. Introduction

Colorectal cancer (CRC) is among the most common malignancies worldwide and a leading cause of cancer-related death, with an estimated 1.9 million new cases and more than 900,000 deaths in 2022 [1]. The burden is projected to rise further over the coming decades [2]. For patients whose disease has not spread to distant organs, surgical resection remains the cornerstone of curative treatment.

In Saudi Arabia, CRC is the most commonly diagnosed cancer in men and one of the most common cancers in women, and its incidence has risen steadily over recent decades [3]. A notable feature of the local setting is delayed presentation, with a substantial proportion of patients diagnosed at an advanced stage. This pattern may be partly attributable to the absence of an established national screening program [4]. Reported five-year survival rates remain lower than those observed in many Western countries [5]. These observations underscore the need for simple, widely available tools to identify patients at higher risk of poor outcomes.

At present, prognosis is estimated mainly from the tumor–node–metastasis (TNM) stage [6]. Although staging is essential, patients with the identical stage may experience markedly different outcomes, which has prompted a search for additional markers that are inexpensive, easy to obtain, and able to provide information beyond tumor anatomy alone.

Increasing evidence indicates that the host inflammatory and nutritional state influences tumor growth and progression [7,8]. Greater systemic inflammation and poorer nutrition are both associated with worse outcomes and can be assessed using routine blood tests [9,10]. Several composite immunonutritional indices have been investigated in CRC, including the controlling nutritional status score and the geriatric nutritional risk index [11,12]. The most extensively studied is the Prognostic Nutritional Index (PNI), described by Onodera and colleagues in 1984 and calculated from serum albumin and the absolute lymphocyte count [13,14,15]. A parallel family of composite scores combines serum albumin with a ratio of circulating leukocyte populations, most commonly the neutrophil-to-lymphocyte ratio (NLR) or the lymphocyte-to-monocyte ratio (LMR) [16,17].

Two issues have received little attention. The first is whether the prognostic effect of these indices is constant across follow-up. The hazard ratios routinely reported assume that it is, yet this assumption is seldom tested, and its failure would alter how the indices should be used in practice. The second is whether the thresholds used to dichotomize them are transferable. Cut-off values derived in East Asian and European cohorts are frequently applied elsewhere without confirming that the underlying distributions are comparable, and recent reviews identify this inconsistency as the principal obstacle to clinical adoption [18].

We therefore examined a single-center Saudi cohort of patients undergoing curative resection for stage I–III colorectal adenocarcinoma, a setting in which a substantial minority present as emergencies. We assessed whether the preoperative PNI and a composite albumin–NLR score independently predict overall survival (OS) and disease-free survival (DFS), which of their constituent components carries the prognostic signal, whether the effect persists throughout follow-up, and how published cut-off values perform when applied to this population.

## 2. Patients and Methods

### 2.1. Study Design and Population

This single-center retrospective cohort study included 316 patients who underwent curative surgical resection for stage I–III colorectal adenocarcinoma at King Abdulaziz University Hospital, Jeddah, Saudi Arabia, between January 2013 and December 2022. Adult patients (≥18 years) with histologically confirmed colorectal adenocarcinoma who underwent curative-intent resection were eligible. Patients with non-adenocarcinoma histology, de novo stage IV (metastatic) disease at diagnosis, local excision only, or incomplete clinical, laboratory, or follow-up data were excluded. Both colon and rectal tumors were included, with rectosigmoid tumors classified as rectal. Elective and emergency resections were both included. One patient with signet-ring cell carcinoma was retained as an adenocarcinoma variant.

We reviewed patient charts for recurrence or death during follow-up. Both local and distant recurrences were counted, and the first date on which recurrence was documented on imaging or in the progress notes was recorded. Patients without documented recurrence were treated as recurrence-free at the date of last contact. Lymphovascular invasion (LVI) was recorded for all 316 patients from the pathology reports.

### 2.2. Immunonutritional Indices

Neutrophil, lymphocyte and monocyte counts, along with serum albumin, were obtained from routine preoperative blood tests performed within one month before resection and processed in the institution’s accredited clinical laboratory using standard automated hematology and biochemistry analyzers. The PNI was calculated according to the original formula of Onodera and colleagues as 10 × albumin (g/dL) + 0.005 × lymphocyte count (per mm^3^) [13]. A composite albumin–NLR score was defined as follows: a score of 0 was assigned to patients with albumin > 40 g/L and NLR ≤ 3; a score of 1 when either parameter was abnormal; and a score of 2 when both were abnormal. One patient had a lymphocyte count of zero, leaving the NLR undefined; this patient was classified as abnormal for the NLR component. For clinical interpretation, the PNI was additionally dichotomized at the cohort median.

In a post hoc analysis, the LMR-based systemic inflammation score of Chang and colleagues [17] and its modified form were also computed, using the published thresholds of albumin ≥ 40 g/L with LMR ≥ 4.44 and ≥3.4 respectively.

### 2.3. Endpoints and Statistical Analysis

OS was defined as the interval from surgery to death from any cause or last follow-up; DFS as the interval from surgery to first recurrence or death. The PNI was the prespecified primary marker and was analyzed as a continuous variable; the albumin–NLR score was the secondary marker and was analyzed as an ordinal variable (0/1/2).

The proportional hazards assumption was assessed for every covariate using scaled Schoenfeld residuals. Because the PNI effect appeared to attenuate over follow-up, an exploratory model split follow-up at 36 months, a cut-point chosen as the approximate midpoint of follow-up and because most recurrences after resection occur within three years. The PNI and the albumin–NLR score share serum albumin, so they were entered into separate models with identical covariates to avoid collinearity and unstable coefficients.

The proportional hazards assumption was assessed for every covariate using scaled Schoenfeld residuals, and a secondary model split follow-up at 36 months to estimate separate PNI coefficients for each period. To identify which constituent carried the prognostic signal, albumin, absolute lymphocyte count, absolute neutrophil count and the NLR were each entered individually into the same adjusted model. Incremental prognostic value was assessed by comparing a clinical model (age, stage, ASA class, emergency presentation, tumor site, neoadjuvant therapy, adjuvant chemotherapy, LVI) with the same model plus the PNI, using Harrell’s C-index and a likelihood-ratio test, with 95% confidence intervals for the C-index and for the change in C-index obtained from 1000 bootstrap resamples. Transportability was examined by applying published PNI thresholds to this cohort and comparing the resulting classification and survival separation with a locally derived threshold. A sensitivity analysis restricted the cohort to patients undergoing upfront surgery, since preoperative laboratory values in neoadjuvant-treated patients are measured after chemoradiotherapy.

A two-sided *p* < 0.05 was considered significant. Reporting followed the STROBE statement [19] and the REMARK recommendations for tumor marker prognostic studies [20]: the completed checklists are provided as Supplementary Materials. Analyses were performed using IBM SPSS Statistics version 31 (IBM Corp., Armonk, NY, USA) and Python 3 (lifelines 0.30.3). Descriptive statistics, Kaplan–Meier estimates, log-rank tests, and Cox models were computed in SPSS; scaled Schoenfeld residual tests, Harrell’s C-index with bootstrap confidence intervals, and the time-split model in counting-process format were computed in lifelines.

## 3. Results

### 3.1. Patient Characteristics

**Figure 1:**
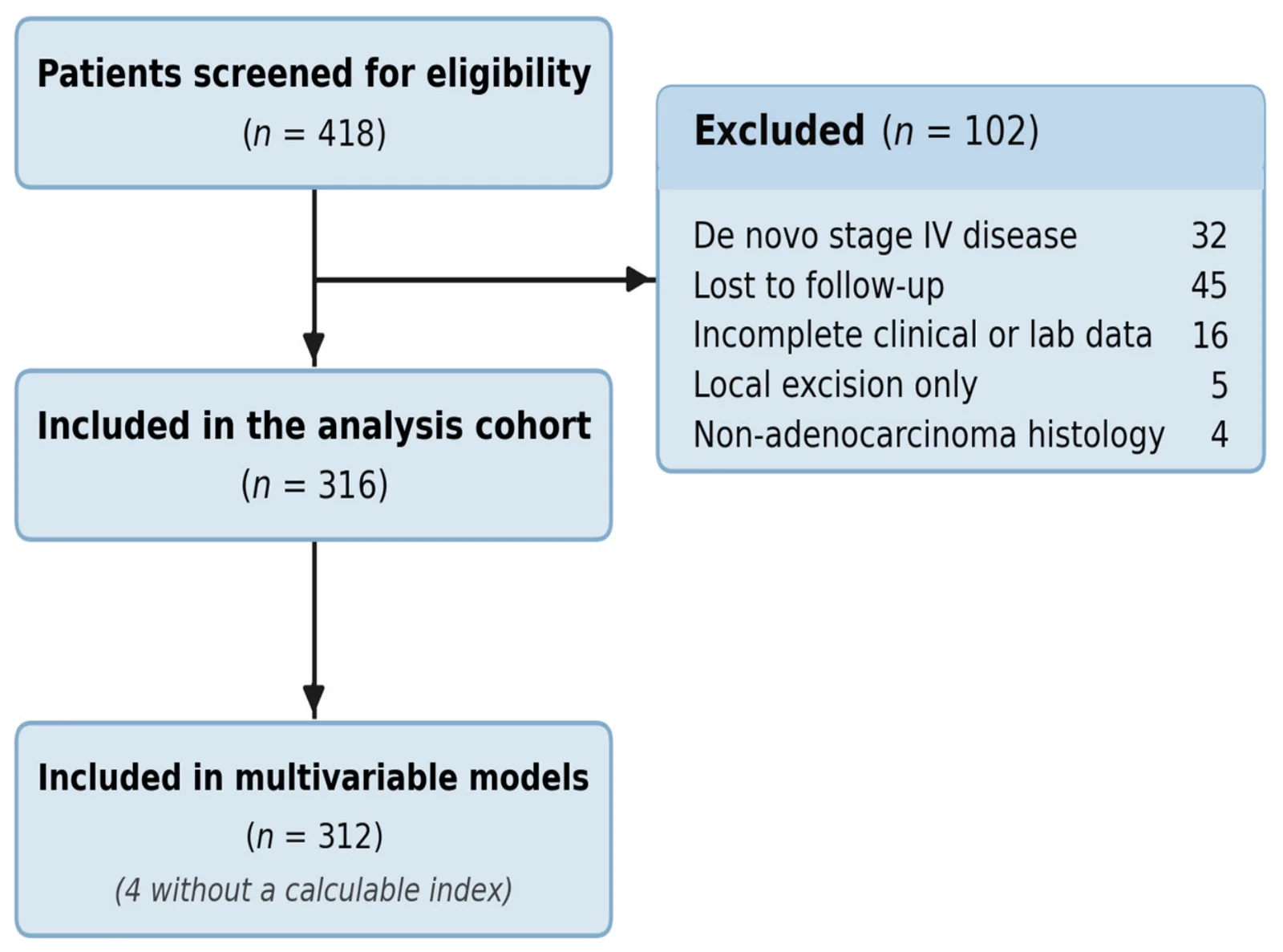
Participant flow, with reasons for exclusion.

**Figure 2:**
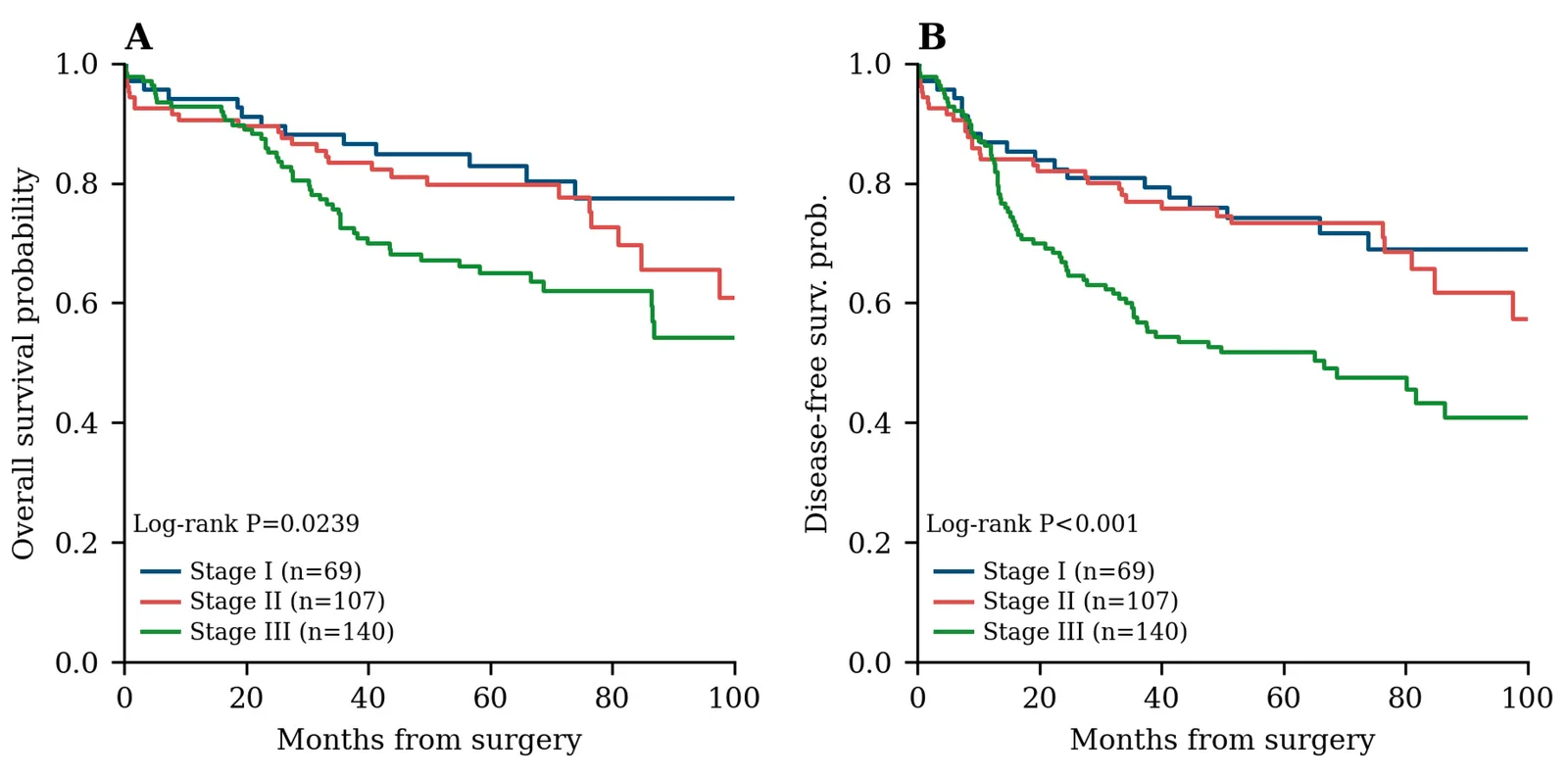
Kaplan–Meier survival curves by tumor stage for (**A**) overall survival and (**B**) disease-free survival.

Of 418 patients screened, 102 were excluded and 316 formed the analysis cohort (Figure 1). Baseline characteristics are summarized in Table 1. Notably, 48 patients (15.2%) underwent emergency resection, neoadjuvant therapy was concentrated among rectal tumors (44/121, 36.4% versus 5/195, 2.6% of colon tumors), and lymphovascular invasion was present in 63 (19.9%). Survival differed significantly by stage (Figure 2). Over a median follow-up of 58.1 months (67.4 months by reverse Kaplan–Meier), 88 patients (27.8%) died, 83 (26.3%) developed recurrence, and 124 (39.2%) experienced a DFS event.

### 3.2. Distribution and Correlates of the Immunonutritional Indices

The median PNI was 42.83 (IQR 37.94–48.67). Against the thresholds proposed in the original description of the index, 119 patients (38.1%) fell above 45, 87 (27.9%) between 40 and 45, and 106 (34.0%) below 40 [13]. The albumin–NLR score distribution was 41 (13.0%) with a score of 0, 181 (57.3%) with a score of 1, and 90 (28.5%) with a score of 2; the score could not be calculated in 4 patients owing to incomplete laboratory data (Table 1).

The components underlying these scores were distributed differently from the source populations in which their thresholds were established. Serum albumin was below 40 g/L in 75.6% of patients, and the median LMR was 2.68 (IQR 1.93–3.85), with 82.6% of values falling below the published threshold of 4.44. Consequently the LMR-based systemic inflammation score assigned 200 of 312 evaluable patients (64.1%) to the highest-risk category and only 14 (4.5%) to the lowest.

The PNI was largely independent of established prognostic variables (Table 2). It did not differ across tumor stage (median 43.60, 43.20 and 42.60 for stages I, II and III; *p* = 0.896), by lymphovascular invasion status (*p* = 0.508), ASA class (*p* = 0.062), sex (*p* = 0.375) or receipt of adjuvant chemotherapy (*p* = 0.981). It was lower in patients presenting as emergencies (37.85 versus 43.40; *p* = 0.001), in those with rectal tumors (*p* = 0.021) and in those receiving neoadjuvant therapy (*p* = 0.047).

### 3.3. Univariable Analysis

Univariable Cox regression (Table 3) showed that older age, advanced stage, ASA class ≥ 3, emergency presentation, neoadjuvant therapy, LVI, lower PNI and higher albumin–NLR score were each associated with poorer OS and DFS. Sex, tumor site and adjuvant chemotherapy were not significantly associated with either outcome. Each one-unit increase in the PNI was associated with better OS (HR 0.938, 95% CI 0.914–0.963; *p* < 0.001), and each one-level increase in the albumin–NLR score with poorer OS (HR 1.656, 1.156–2.374; *p* = 0.006).

### 3.4. Multivariable Analysis

In the multivariable model (Table 4), the PNI remained an independent predictor of both OS (aHR 0.958, 95% CI 0.929–0.987; *p* = 0.004) and DFS (aHR 0.964, 0.940–0.988; *p* = 0.004). Emergency presentation and neoadjuvant therapy were independently associated with poorer outcomes, and LVI was strongly associated with DFS (aHR 2.284, 1.479–3.528; *p* < 0.001) but not with OS (aHR 1.454, 0.849–2.492; *p* = 0.173).

The albumin–NLR score, examined in a separate model with identical covariates, was not independently associated with OS (aHR 1.146, 0.780–1.683; *p* = 0.488) and was only marginally associated with DFS (aHR 1.412, 1.022–1.950; *p* = 0.037), despite significance on univariable analysis for both endpoints.

**Figure 3:**
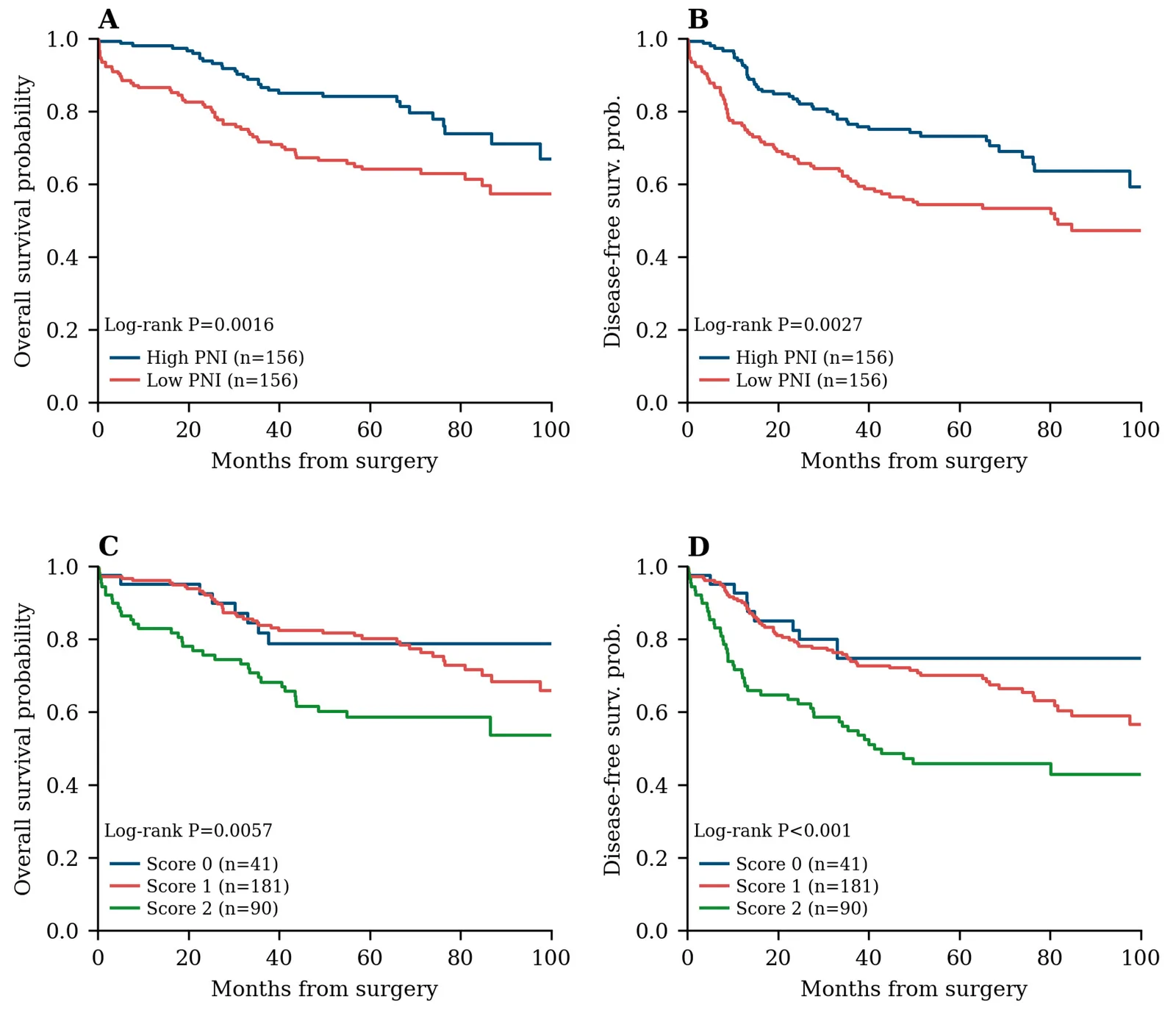
Kaplan–Meier survival curves according to the prognostic nutritional index, dichotomized at the cohort median, for (**A**) overall survival and (**B**) disease-free survival and according to the albumin–NLR score for (**C**) overall survival and (**D**) disease-free survival.

When dichotomized at the cohort median, the PNI identified marked differences in survival. The 5-year OS rate was 84.2% in the high-PNI group compared with 64.1% in the low-PNI group, and the 5-year DFS rate was 73.3% versus 54.4% (Figure 3).

### 3.5. Contribution of Individual Components and Alternative Scores

Entered individually into the same adjusted model, serum albumin was independently associated with both endpoints (OS aHR 0.938 per g/L, 95% CI 0.906–0.970, *p* < 0.001; DFS aHR 0.950, 0.924–0.978, *p* < 0.001). None of the cellular components retained any association: absolute lymphocyte count (OS aHR 1.085, *p* = 0.56), absolute neutrophil count (OS aHR 0.999, *p* = 0.98) and the NLR (OS aHR 0.997, *p* = 0.85) were all null (Table 5).

Emergency presentation altered these components selectively. Mean albumin fell from 35.2 g/L in elective patients to 30.2 g/L in emergency patients (*p* < 0.001) and the mean neutrophil count rose from 3.96 to 8.07 × 10^9^/L (*p* = 0.002), whereas the mean lymphocyte count was unchanged (1.74 versus 1.78; *p* = 0.76).

The same pattern applied to the LMR-based systemic inflammation score and its modified form, examined post hoc: neither was significantly associated with either endpoint even before adjustment (OS HR 1.504, 0.990–2.283, *p* = 0.056; DFS HR 1.399, 0.994–1.967, *p* = 0.054), and both were null after adjustment (Table 5).

### 3.6. Proportional Hazards Assessment and Time-Dependence

Scaled Schoenfeld residuals showed no significant violation of the proportional hazards assumption for the PNI (OS *p* = 0.075; DFS *p* = 0.085). Neoadjuvant therapy violated the assumption for DFS (*p* = 0.004) and adjuvant chemotherapy marginally so (*p* = 0.033); results were unchanged when the DFS model was additionally stratified by neoadjuvant status.

Splitting follow-up at 36 months nonetheless showed that the prognostic effect of the PNI was confined to the early period. For OS, the aHR was 0.944 (0.913–0.976; *p* < 0.001) within the first 36 months and 1.003 (0.947–1.063; *p* = 0.91) thereafter; for DFS, the corresponding estimates were 0.956 (0.931–0.982; *p* = 0.001) and 1.002 (0.948–1.060; *p* = 0.93) (Table 6).

### 3.7. Incremental Value and Sensitivity Analyses

Adding the PNI to the clinical model improved discrimination modestly. For OS, Harrell’s C-index rose from 0.738 (95% CI 0.696–0.799) to 0.757 (0.715–0.818), and for DFS from 0.698 (0.663–0.753) to 0.717 (0.678–0.772). The likelihood-ratio test favored inclusion of the PNI for both endpoints (OS χ^2^ = 8.96, *p* = 0.003; DFS χ^2^ = 8.25, *p* = 0.004), but the bootstrap confidence interval for the change in C-index included zero (OS +0.020, 95% CI −0.001 to +0.050; DFS +0.019, −0.000 to +0.046), so the gain in discrimination should be regarded as small and imprecisely estimated. Restricting the analysis to the 264 patients undergoing upfront surgery strengthened the PNI effect (OS aHR 0.944, 0.914–0.975, *p* < 0.001; DFS aHR 0.957, 0.932–0.983, *p* = 0.001).

### 3.8. Performance of Published Cut-Offs

Seven thresholds were applied to the cohort (Table 7). Discrimination declined monotonically as the threshold rose above the cohort distribution, from a 5-year overall survival difference of 82.1% versus 58.9% (*p* < 0.001) at a threshold of 40, to *p* = 0.025 at the receiver operating characteristic–derived value of 47.5 [21], to no significant disease-free survival difference (*p* = 0.051) at 48. Cut-offs derived in populations with higher PNI distributions performed least well.

## 4. Discussion

In this single-center Saudi cohort of patients who underwent curative resection for stage I–III CRC, the PNI was a robust independent predictor of both OS and DFS, remaining significant after adjustment for tumor stage, ASA class, emergency presentation, tumor site, neoadjuvant therapy, adjuvant chemotherapy and lymphovascular invasion. In contrast, although the composite albumin–NLR score was significantly associated with survival in the univariable analysis, it was not an independent prognostic factor after multivariable adjustment.

Several clinical factors may explain this finding. Emergency presentation was a powerful independent predictor of poor survival [23,24] and was closely associated with a higher albumin–NLR score because emergency patients were more likely to have hypoalbuminemia and an elevated NLR. Neoadjuvant therapy, administered predominantly to patients with rectal tumors, was also associated with poorer survival and is known to alter neutrophil and lymphocyte counts, thereby influencing these indices [25]. Consequently, the albumin–NLR score may have reflected acute illness and treatment-related effects rather than an independent biological signal.

### 4.1. The Contribution of Albumin

The component analysis supports this interpretation. When entered individually into the same adjusted model, serum albumin retained a strong independent association with both endpoints, whereas the lymphocyte count, the neutrophil count and the NLR were each null. The composite therefore appears to add little beyond its albumin term. Notably, the PNI did not vary with tumor stage or lymphovascular invasion, indicating that it captures host physiological state rather than tumor burden, which is consistent with its retaining independence after stratification by stage.

The mechanism is visible in the component data. Emergency presentation depressed albumin and elevated neutrophils while leaving lymphocytes unchanged, so a score constructed from albumin and a neutrophil-containing ratio will tend to track acute physiological derangement. This may help explain a long-standing inconsistency in the literature, in which inflammation-based composites appear independently prognostic in some cohorts and not others. Where surgical urgency is unmeasured or unadjusted, an apparent independent association may partly reflect it. Studies of these indices in mixed elective and emergency cohorts would therefore benefit from adjusting for urgency and those restricted to elective surgery from stating so, since the two settings are not equivalent.

### 4.2. A Time-Limited Effect

In an exploratory analysis splitting follow-up at 36 months, the prognostic effect of the PNI was confined to the early period and absent thereafter for both endpoints. Formal testing showed no significant violation of the proportional hazards assumption, so this should be regarded as hypothesis-generating. It is nonetheless consistent with external longitudinal data, in which cancer-related deaths clustered earlier in follow-up and the PNI declined most steeply during that period [26]. An effect concentrated in the first three postoperative years is more readily explained by perioperative physiological reserve, tolerance of adjuvant therapy and early treatment-related attrition than by tumor biology, and these are potentially modifiable. The preoperative PNI may therefore be most useful for targeting nutritional optimization, prehabilitation and intensified early surveillance.

### 4.3. Performance of Imported Thresholds

The distribution of the PNI in this cohort differed materially from those in which the commonly used thresholds were derived. A threshold of 47.5, obtained by receiver operating characteristic analysis in a Turkish cohort of 489 patients [21], classified over 70% of our patients as low-PNI and separated survival less effectively than a locally derived value. The same difficulty applies more severely to the LMR-based systemic inflammation score, which in our post hoc analysis was not associated with either endpoint: with 82.6% of patients below the published LMR threshold of 4.44, the score assigned almost two-thirds of the cohort to a single category and retained little capacity to discriminate. Even the original thresholds of the PNI itself, at 40 and 45, placed a third of this cohort in the range once regarded as a contraindication to resection and anastomosis [13], which suggests that these values reflect the nutritional profile of the population in which they were derived rather than a universal biological boundary.

These observations extend beyond the present cohort. Recent regional reviews identify inconsistent cut-off definitions as the principal obstacle to clinical adoption of these indices [18], and a meta-analysis of inflammatory indices in locally advanced rectal cancer found that the cut-off value and the method of its derivation partly explained between-study heterogeneity [27]. Our data provide a concrete illustration: thresholds are population-dependent, and applying them without first examining the local distribution can produce both misclassification and loss of discrimination.

### 4.4. Comparison with the Literature

Our findings regarding the PNI are consistent with the published literature [28,29]. A meta-analysis of 43 studies involving 19,214 patients with CRC reported a pooled hazard ratio for OS of 1.89 for low versus high PNI, although its pooled estimates for disease-free and progression-free survival did not reach significance [30]. Our findings agree in direction for OS and differ in showing an independent association with DFS; the estimates are not directly comparable, since ours are expressed per unit of a continuous variable. That meta-analysis also identified the PNI cut-off as a source of between-study heterogeneity, consistent with our own threshold analysis. The most directly comparable study is a Turkish series of 489 patients undergoing curative CRC resection, in which a low PNI independently predicted poorer OS and DFS alongside stage, age and perineural invasion [21]; our results agree on independence and direction while differing on the transportability of the threshold. In metastatic disease, a Turkish cohort of 253 patients reported longer OS above a PNI of 46.6 [22], and a series of 637 rectal cancer patients found albumin, the CRP-to-albumin ratio, the NLR and the systemic immune-inflammation index all associated with survival [31].

Our result for the composite score differs from that of a European series of 616 patients undergoing curative resection for stage I–III disease, in which inflammation-based indices retained independent prognostic value after adjustment [16]. That cohort was drawn from elective practice, whereas 15.2% of our patients underwent emergency resection. Because emergency presentation depressed albumin and elevated neutrophils in our data and was itself a strong independent predictor of survival, the discrepancy is what would be expected if the independence of these composites depends on the case mix in which they are evaluated. This is a testable explanation rather than a conflict of findings and could be examined directly in any cohort that records urgency of presentation.

Regional data on inflammation-based markers in resected CRC remain sparse. The closest Arab-region comparator is a Jordanian cohort of 285 patients undergoing elective colectomy, in which a high NLR predicted worse cancer-specific survival after adjustment while the platelet-to-lymphocyte ratio showed no association [32]—a pattern of partial and selective survival of inflammatory ratios after adjustment that resembles our own. Iranian groups have reported on newer composites such as the CRP–albumin–lymphocyte index in large multicenter cohorts [33]. To our knowledge, no study of immunonutritional indices in resected CRC has been reported from the Arab Gulf, and none from the wider region has examined whether imported thresholds transport.

### 4.5. Strengths and Limitations

The strengths of this study include complete ascertainment of lymphovascular invasion and of adjuvant and neoadjuvant therapy in all patients, adjustment for surgical urgency in a cohort with a substantial emergency caseload, formal testing of the proportional hazards assumption, and quantification of incremental prognostic value rather than statistical significance alone.

Several limitations apply. The retrospective, single-center design may limit generalizability and introduce selection and information bias. The dichotomized PNI threshold was derived from the study cohort and requires external validation, although the primary continuous analysis does not depend on it. The analyses of the LMR-based score, the 36-month split and cut-off performance were exploratory. Cause of death was not recorded, so competing-risks models could not be fitted, and cancer-specific endpoints should be collected prospectively. Tumor grade, resection margin status, perineural invasion, nodal yield and mismatch repair status were unavailable. Finally, recurrence data were collected retrospectively, which may have affected DFS ascertainment. These findings should be regarded as hypothesis-strengthening rather than definitive.

## 5. Conclusions

In a Saudi cohort undergoing curative resection for stage I–III colorectal cancer, the preoperative PNI was an independent predictor of overall and disease-free survival, subject to three qualifications that bear on how it should be used. Its prognostic signal was carried by serum albumin rather than by any inflammatory component; it operated only within the first three postoperative years; and it was sensitive to the provenance of the threshold applied, with published cut-offs classifying the majority of this population as high-risk. Composite inflammation-based scores lost independence once surgical urgency, neoadjuvant therapy and lymphovascular invasion were modeled. Preoperative albumin-based assessment may be most useful for identifying patients at elevated risk of early death and recurrence, in whom nutritional optimization and intensified early surveillance could be worthwhile, although its incremental discriminatory gain over standard clinical variables was small. Locally derived thresholds should be established before these indices are applied in new populations.

## Figures and Tables

**Table 1:** Baseline characteristics and preoperative immunonutritional indices (*n* = 316).

Characteristic	Value
Age, median (IQR), years	60 (51–70)
Male	179 (56.6%)
Female	137 (43.4%)
BMI, median	26.8
Stage I	69 (21.8%)
Stage II	107 (33.9%)
Stage III	140 (44.3%)
ASA class 1	12 (3.8%)
ASA class 2	170 (53.8%)
ASA class 3	128 (40.5%)
ASA class 4	5 (1.6%)
ASA class 5	1 (0.3%)
Elective surgery	268 (84.8%)
Emergency surgery	48 (15.2%)
Colon	195 (61.7%)
Rectum/rectosigmoid	121 (38.3%)
Neoadjuvant therapy	49 (15.5%)
colon	5/195 (2.6%)
rectum	44/121 (36.4%)
Adjuvant chemotherapy	140 (44.3%)
Lymphovascular invasion present	63 (19.9%)
Deaths	88 (27.8%)
Recurrences	83 (26.3%)
DFS events	124 (39.2%)
Median follow-up, months	58.1 (reverse Kaplan–Meier 67.4)
PNI, median (IQR)	42.83 (37.94–48.67)
Albumin–NLR score = 0	41 (13.0%)
Albumin–NLR score = 1	181 (57.3%)
Albumin–NLR score = 2	90 (28.5%)

IQR, interquartile range; ASA, American Society of Anesthesiologists; BMI, body mass index; DFS, disease-free survival; PNI, prognostic nutritional index; NLR, neutrophil-to-lymphocyte ratio. The albumin–NLR score was not calculable in 4 patients owing to incomplete laboratory data; percentages are of the full cohort. One patient with a lymphocyte count of zero (NLR undefined) was classified as abnormal for the NLR component.

**Table 2:** Distribution of the prognostic nutritional index across standard prognostic variables (*n* = 312).

Variable	Category	PNI, Median (IQR)	*p*
Stage	I (*n* = 68)	43.60 (37.79–47.90)	0.896
	II (*n* = 107)	43.20 (37.40–48.92)	
	III (*n* = 137)	42.60 (38.45–48.65)	
ASA class	1–2 (*n* = 181)	43.20 (38.55–49.50)	0.062
	≥3 (*n* = 131)	41.90 (37.15–47.73)	
Presentation	Elective (*n* = 265)	43.40 (38.45–49.10)	0.001
	Emergency (*n* = 47)	37.85 (33.80–45.40)	
Tumor site	Colon (*n* = 193)	44.00 (37.90–49.70)	0.021
	Rectum/rectosigmoid (*n* = 119)	41.65 (37.95–46.45)	
Lymphovascular invasion	Absent (*n* = 250)	42.83 (37.82–48.65)	0.508
	Present (*n* = 62)	42.80 (38.45–49.25)	
Neoadjuvant therapy	No (*n* = 264)	43.25 (37.99–49.38)	0.047
	Yes (*n* = 48)	40.75 (37.38–45.58)	
Adjuvant chemotherapy	No (*n* = 175)	43.20 (37.83–48.70)	0.981
	Yes (*n* = 137)	42.65 (38.15–48.65)	
Sex	Female (*n* = 135)	42.80 (37.55–47.67)	0.375
	Male (*n* = 177)	42.95 (38.25–49.45)	

IQR, interquartile range; ASA, American Society of Anesthesiologists. Comparisons by Mann–Whitney U test (two groups) or Kruskal–Wallis test (three groups). Four patients with incomplete laboratory data are excluded.

**Table 3:** Univariable Cox regression for overall and disease-free survival.

Variable	OS HR (95% CI)	OS *p*	DFS HR (95% CI)	DFS *p*
Age (per year)	1.036 (1.019–1.053)	<0.001	1.018 (1.004–1.032)	0.013
Male sex	0.875 (0.575–1.332)	0.534	0.891 (0.624–1.271)	0.525
Stage (per level)	1.478 (1.109–1.970)	0.008	1.593 (1.242–2.042)	<0.001
ASA ≥ 3	2.089 (1.371–3.183)	<0.001	1.853 (1.300–2.642)	<0.001
Emergency presentation	2.637 (1.625–4.280)	<0.001	2.110 (1.367–3.257)	<0.001
Rectal/rectosigmoid site	0.882 (0.571–1.361)	0.570	0.905 (0.628–1.304)	0.591
Neoadjuvant therapy	2.015 (1.232–3.293)	0.005	1.794 (1.170–2.751)	0.007
Adjuvant chemotherapy	0.857 (0.561–1.309)	0.476	1.046 (0.734–1.491)	0.804
Lymphovascular invasion	1.823 (1.139–2.918)	0.012	2.640 (1.809–3.852)	<0.001
PNI (continuous)	0.938 (0.914–0.963)	<0.001	0.955 (0.933–0.976)	<0.001
Albumin–NLR score (ordinal)	1.656 (1.156–2.374)	0.006	1.683 (1.243–2.279)	<0.001

HR, hazard ratio; OS, overall survival; DFS, disease-free survival; CI, confidence interval. PNI per one-unit increase; albumin–NLR score per one-level increase; stage per one-level increase.

**Table 4:** Multivariable Cox regression for overall and disease-free survival.

Variable	OS aHR (95% CI)	OS *p*	DFS aHR (95% CI)	DFS *p*
PNI (per unit)	0.958 (0.929–0.987)	0.004	0.964 (0.940–0.988)	0.004
Age (per year)	1.033 (1.017–1.050)	<0.001	1.015 (1.001–1.029)	0.037
Male sex	0.983 (0.631–1.531)	0.940	0.905 (0.622–1.316)	0.601
Emergency presentation	2.229 (1.260–3.943)	0.006	1.695 (1.033–2.783)	0.037
Rectal/rectosigmoid site	0.693 (0.369–1.304)	0.256	0.721 (0.437–1.188)	0.199
Neoadjuvant therapy	2.998 (1.506–5.965)	0.002	2.252 (1.282–3.955)	0.005
Adjuvant chemotherapy	0.709 (0.411–1.223)	0.216	0.661 (0.415–1.053)	0.082
Lymphovascular invasion	1.454 (0.849–2.492)	0.173	2.284 (1.479–3.528)	<0.001

aHR, adjusted hazard ratio. Model stratified by stage and ASA class; *n* = 312, with 87 deaths and 123 DFS events.

**Table 5:** Univariable and adjusted associations for the individual index components and for alternative composite scores.

Variable	Endpoint	Univariable HR (95% CI)	*p*	Adjusted HR (95% CI)	*p*
Albumin (per g/L)	OS	0.920 (0.894–0.948)	<0.001	0.938 (0.906–0.970)	<0.001
	DFS	0.943 (0.919–0.968)	<0.001	0.950 (0.924–0.978)	<0.001
Lymphocytes (per 10^9^/L)	OS	0.928 (0.719–1.198)	0.567	1.085 (0.825–1.427)	0.558
	DFS	0.911 (0.735–1.130)	0.397	1.030 (0.808–1.312)	0.813
Neutrophils (per 10^9^/L)	OS	1.044 (1.015–1.074)	0.003	0.999 (0.965–1.035)	0.976
	DFS	1.034 (1.007–1.062)	0.013	0.995 (0.964–1.027)	0.763
NLR (per unit)	OS	1.009 (0.988–1.031)	0.407	0.997 (0.963–1.031)	0.854
	DFS	1.006 (0.985–1.027)	0.575	0.996 (0.965–1.028)	0.817
Systemic inflammation score	OS	1.504 (0.990–2.283)	0.056	1.084 (0.693–1.696)	0.725
	DFS	1.399 (0.994–1.967)	0.054	1.275 (0.884–1.838)	0.193
Modified systemic inflammation score	OS	1.374 (0.975–1.936)	0.070	1.027 (0.705–1.494)	0.891
	DFS	1.372 (1.030–1.826)	0.030	1.193 (0.875–1.625)	0.265

HR, hazard ratio; OS, overall survival; DFS, disease-free survival; NLR, neutrophil-to-lymphocyte ratio. Each variable was entered individually; adjusted models used the covariates and stratification of Table 4. The systemic inflammation score and its modified form are based on albumin and the lymphocyte-to-monocyte ratio and were examined post hoc. Score distributions: systemic inflammation score 0/1/2 = 14/98/200; modified score 0/1/2 = 33/115/164.

**Table 6:** Time-split analysis of the PNI effect, with follow-up divided at 36 months.

Endpoint	0–36 Months, aHR (95% CI)	*p*	>36 Months, aHR (95% CI)	*p*
OS	0.944 (0.913–0.976)	<0.001	1.003 (0.947–1.063)	0.909
DFS	0.956 (0.931–0.982)	0.001	1.002 (0.948–1.060)	0.930

Covariates and stratification are as in Table 4.

**Table 7:** Performance of published prognostic nutritional index cut-offs applied to this cohort.

Cut-Off	Low-PNI, *n* (%)	5-y OS, High vs. Low	*p*	5-y DFS, High vs. Low	*p*	Source Population
40.00	106 (34.0%)	82.1% vs. 58.9%	<0.001	70.5% vs. 50.9%	0.004	Onodera, lower bound [13]
42.83	156 (50.0%)	84.2% vs. 64.1%	0.002	73.3% vs. 54.4%	0.003	This cohort (median)
44.00	171 (54.8%)	85.0% vs. 65.2%	<0.001	74.2% vs. 55.2%	0.001	Japanese CRC cohorts
45.00	190 (60.9%)	85.5% vs. 66.7%	0.002	74.7% vs. 56.6%	0.003	Onodera, upper bound [13]
46.60	213 (68.3%)	85.4% vs. 68.7%	0.010	76.3% vs. 57.8%	0.005	Turkish metastatic CRC [22]
47.50	221 (70.8%)	84.1% vs. 69.8%	0.025	74.2% vs. 59.3%	0.023	Turkish CRC, ROC-derived [21]
48.00	224 (71.8%)	83.4% vs. 70.2%	0.048	73.2% vs. 59.9%	0.051	Chinese CRC cohorts

ROC, receiver operating characteristic. Corresponding DFS log-rank comparisons were *p* = 0.023 at a cut-off of 47.5 and *p* = 0.003 at the cohort median.

## Data Availability

The de-identified dataset supporting the conclusions of this article is available from the corresponding author on reasonable request.

## Supplementary Materials

The following supporting information can be downloaded at https://www.mdpi.com/article/10.3390/curroncol33090496/s1: File S1, containing the completed STROBE and REMARK reporting checklists.
