# Supplementary material for: Preoperative Immunonutritional Indices in Colorectal Cancer: The Contribution of Albumin, Time-Dependence of Effect, and Threshold Transportability in a Saudi Cohort
Source: Curr Oncol. 2026 Aug 22;33(9):496. doi: 10.3390/curroncol33090496 (PMC13605432; doi:10.3390/curroncol33090496)
Supplement: Supplementary file 1 [file curroncol-33-00496-s001.zip › curroncol-4507481-supplementary.pdf]

Supplementary File S1: Completed STROBE and REMARK reporting checklists

STROBE Statement—checklist of items that should be included in reports of observational studies

	Item No.	Recommendation	Page No.	Relevant text from manuscript
**Title and abstract**	1	(*a*) Indicate the study's design with a commonly used term in the title or the abstract	3	Abstract: "In this retrospective cohort study we analyzed 316 patients undergoing curative resection for stage I–III colorectal adenocarcinoma…"
		(*b*) Provide in the abstract an informative and balanced summary of what was done and what was found	3	Abstract gives objective, design, setting, cohort size, covariates, principal effect estimates with 95% CIs, and the limits of the incremental-value finding.
**Introduction**				
Background/rationale	2	Explain the scientific background and rationale for the investigation being reported	4	Introduction, paragraphs 1–4: burden of CRC, Saudi epidemiology and late presentation, limits of TNM staging, and the immunonutritional index literature.
Objectives	3	State specific objectives, including any prespecified hypotheses	4	Introduction, final two paragraphs: the two unresolved issues (constancy of effect over follow-up; transferability of thresholds) and the four stated objectives.
**Methods**				
Study design	4	Present key elements of study design early in the paper	5	Section 2.1: "This single-center retrospective cohort study included 316 patients…"
Setting	5	Describe the setting, locations, and relevant dates, including periods of recruitment, exposure, follow-up, and data collection	5	Section 2.1: King Abdulaziz University Hospital, Jeddah, Saudi Arabia; January 2013 to December 2022. Follow-up summarized in Section 3.1.
Participants	6	(*a*) *Cohort study*—Give the eligibility criteria, and the sources and methods of selection of participants. Describe methods of follow-up<br>*Case-control study*—Give the eligibility criteria, and the sources and methods of case ascertainment and control selection. Give the rationale for the choice of cases and controls<br>*Cross-sectional study*—Give the eligibility criteria, and the	5	Section 2.1: eligibility (adults ≥18 years, histologically confirmed colorectal adenocarcinoma, curative-intent resection) and exclusions (non-adenocarcinoma histology, de novo stage IV, local excision only, incomplete data).

		sources and methods of selection of participants		
		(*b*) *Cohort study*—For matched studies, give matching criteria and number of exposed and unexposed<br>*Case-control study*—For matched studies, give matching criteria and the number of controls per case	—	Not applicable — unmatched cohort.
Variables	7	Clearly define all outcomes, exposures, predictors, potential confounders, and effect modifiers. Give diagnostic criteria, if applicable	5–6	Sections 2.2 and 2.3: outcomes (OS, DFS) defined; markers (PNI, albumin–NLR score) defined; confounders listed (age, sex, emergency presentation, tumor site, neoadjuvant therapy, adjuvant chemotherapy, lymphovascular invasion); stage and ASA class used as strata.
Data sources/ measurement	8*	For each variable of interest, give sources of data and details of methods of assessment (measurement). Describe comparability of assessment methods if there is more than one group	5	Section 2.2: routine preoperative blood tests within one month of resection, processed in the institution's accredited clinical laboratory on standard automated hematology and biochemistry analyzers. Section 2.1: lymphovascular invasion from pathology reports; recurrence from imaging and clinic documentation.
Bias	9	Describe any efforts to address potential sources of bias	6, 19	Section 2.3: multivariable adjustment, stratification by variables violating proportional hazards, separate models for collinear composite scores, and a sensitivity analysis restricted to upfront surgery. Section 4.5 discusses selection and information bias.
Study size	10	Explain how the study size was arrived at	6	Section 2.3: "No formal sample size calculation was performed; the cohort comprised all consecutive eligible patients treated during the study period."

Continued on next page

Quantitative variables	11	Explain how quantitative variables were handled in the analyses. If applicable, describe which groupings were chosen and why	6, 9	Section 2.3: PNI analyzed as a continuous variable (primary); albumin–NLR score as an ordinal variable (0/1/2). Section 2.2 and 3.2: PNI additionally dichotomized at the cohort median (42.83) for clinical interpretation only.
Statistical methods	12	(*a*) Describe all statistical methods, including those used to control for confounding	6	Section 2.3: chi-square/Fisher exact, Mann–Whitney U, Kaplan–Meier with log-rank, univariable and multivariable Cox models with stratification.
		(*b*) Describe any methods used to examine subgroups and interactions	6, 14	Section 2.3 and 3.6: interaction with time examined by a prespecified model splitting follow-up at 36 months, with separate PNI coefficients for each period (Table 6). No other subgroup or interaction analyses were performed.
		(*c*) Explain how missing data were addressed	5, 6	Complete-case analysis throughout; no imputation was performed. Patients with incomplete clinical, laboratory or follow-up data were excluded at eligibility (Section 2.1).
		(*d*) *Cohort study*—If applicable, explain how loss to follow-up was addressed<br>*Case-control study*—If applicable, explain how matching of cases and controls was addressed<br>*Cross-sectional study*—If applicable, describe analytical methods taking account of sampling strategy	6	Section 2.3: OS and DFS censored at the date of last follow-up; patients without documented recurrence treated as recurrence-free at last contact (Section 2.1).
		(*e*) Describe any sensitivity analyses	6, 15	Section 2.3 and 3.6: analysis restricted to the 264 patients undergoing upfront surgery; additional stratification of the DFS model by neoadjuvant status; bootstrap confidence intervals for the C-index and for its change.
**Results**				
Participants	13*	(a) Report numbers of individuals at each stage of study—eg numbers potentially eligible, examined for eligibility, confirmed eligible, included in the study, completing follow-up, and analysed	7	Section 3.1: "Of 418 patients screened, 102 were excluded… leaving 316 for analysis." Multivariable models include 312 patients.
		(b) Give reasons for non-participation at each stage	8	Section 3.1: exclusions principally for de novo stage IV disease at diagnosis or incomplete follow-up data.
		(c) Consider use of a flow diagram	8	No flow diagram used; numbers at each stage are re-

				ported in the text of Section 3.1.
Descriptive data	14*	(a) Give characteristics of study participants (eg demographic, clinical, social) and information on exposures and potential confounders	7	Table 1: demographic, clinical and treatment characteristics, and the distribution of the immunonutritional indices.
		(b) Indicate number of participants with missing data for each variable of interest	7, 12	Table 1 footnote: albumin–NLR score not calculable in 4 patients (1.3%). Section 3.2 and Table 2: 312 patients with an available PNI. Table 4: multivariable models based on n = 312, with 87 deaths and 123 DFS events.
		(c) *Cohort study*—Summarise follow-up time (eg, average and total amount)	7	Section 3.1: median follow-up 58.1 months (67.4 months by reverse Kaplan–Meier).
Outcome data	15*	*Cohort study*—Report numbers of outcome events or summary measures over time	7	Section 3.1: 88 deaths (27.8%), 83 recurrences (26.3%), 124 DFS events (39.2%).
		Case-control study—Report numbers in each exposure category, or summary measures of exposure	—	Not applicable — cohort study.
		Cross-sectional study—Report numbers of outcome events or summary measures	—	Not applicable — cohort study.
Main results	16	(*a*) Give unadjusted estimates and, if applicable, confounder-adjusted estimates and their precision (eg, 95% confidence interval). Make clear which confounders were adjusted for and why they were included	11–12	Table 3 (univariable) and Table 4 (multivariable) give hazard ratios with 95% confidence intervals and p-values; Section 2.3 and the Table 4 footnote state which confounders were adjusted for and why.
		(*b*) Report category boundaries when continuous variables were categorized	5, 15	Section 2.2: albumin–NLR score category definitions. Section 3.2: PNI dichotomized at the cohort median of 42.83. Table 7: published cut-offs applied to this cohort.
		(*c*) If relevant, consider translating estimates of relative risk into absolute risk for a meaningful time period	12	Section 3.4: 5-year OS 84.2% versus 64.1% and 5-year DFS 73.3% versus 54.4% for high versus low PNI.

Continued on next page

Other analyses	17	Report other analyses done—eg analyses of subgroups and interactions, and sensitivity analyses	13–15	Sections 3.5–3.7 and Tables 5–7: component analysis, post hoc analysis of LMR-based composite scores, proportional hazards testing, 36-month time split, incremental value with bootstrap intervals, upfront-surgery sensitivity analysis, and performance of published cut-offs.
**Discussion**				
Key results	18	Summarise key results with reference to study objectives	16	Section 4, opening paragraphs, and Section 5: PNI independently prognostic; signal carried by albumin; effect confined to the first 36 months; imported cut-offs misclassify.
Limitations	19	Discuss limitations of the study, taking into account sources of potential bias or imprecision. Discuss both direction and magnitude of any potential bias	19	Section 4.5: retrospective single-center design, data-derived threshold, post hoc analyses, unavailable histopathological and molecular variables, competing risks, retrospective recurrence ascertainment, and the bootstrap interval for the C-index gain including zero.
Interpretation	20	Give a cautious overall interpretation of results considering objectives, limitations, multiplicity of analyses, results from similar studies, and other relevant evidence	16–19	Sections 4.1–4.3 and Section 5 interpret the findings against the stated objectives and acknowledge the modest incremental discrimination.
Generalisability	21	Discuss the generalisability (external validity) of the study results	19	Section 4.5: single-center retrospective design may limit generalizability; Section 4.3 argues thresholds are population-dependent.
**Other information**				
Funding	22	Give the source of funding and the role of the funders for the present study and, if applicable, for the original study on which the present article is based	20	Funding statement: "This research received no external funding."

*Give information separately for cases and controls in case-control studies and, if applicable, for exposed and unexposed groups in cohort and cross-sectional studies.

Note: An Explanation and Elaboration article discusses each checklist item and gives methodological background and published examples of transparent reporting. The STROBE checklist is best used in conjunction with this article (freely available on the Web sites of PLoS Medicine at http://www.plosmedicine.org/, Annals of Internal Medicine at http://www.annals.org/, and Epidemiology at http://www.epidem.com/). Information on the STROBE Initiative is available at www.strobe-statement.org.

REMARK reporting checklist

Item to be reported		Page no.
**INTRODUCTION**		
1	State the marker examined, the study objectives, and any pre-specified hypotheses.<br><br>Reported: Section 1 (Introduction), final two paragraphs: PNI as prespecified primary marker, albumin–NLR score as secondary; four stated objectives.	4
**MATERIALS AND METHODS**		
Patients		
2	Describe the characteristics (e.g., disease stage or co-morbidities) of the study patients, including their source and inclusion and exclusion criteria.<br><br>Reported: Section 2.1 and Table 1: stage I–III colorectal adenocarcinoma, age, sex, ASA class, urgency of presentation, tumor site.	5, 7
3	Describe treatments received and how chosen (e.g., randomized or rule-based).<br><br>Reported: Section 2.1, Section 3.1 and Table 1: elective versus emergency resection, neoadjuvant therapy (49; 15.5%), adjuvant chemotherapy (140; 44.3%); treatment allocated by routine clinical practice.	5, 7
Specimen characteristics		
4	Describe type of biological material used (including control samples) and methods of preservation and storage.<br><br>Reported: Section 2.2: peripheral venous blood collected as part of routine preoperative testing within one month before resection; no archival specimens or control samples used.	5
Assay methods		
5	Specify the assay method used and provide (or reference) a detailed protocol, including specific reagents or kits used, quality control procedures, reproducibility assessments, quantitation methods, and scoring and reporting protocols. Specify whether and how assays were performed blinded to the study endpoint.<br><br>Reported: Section 2.2: processed in the institution's accredited clinical laboratory using standard automated hematology and biochemistry analyzers.	5
Study design		
6	State the method of case selection, including whether prospective or retrospective and whether stratification or matching (e.g., by stage of disease or age) was used. Specify the time period from which cases were taken, the end of the follow-up period, and the median follow-up time.<br><br>Reported: Section 2.1: retrospective, consecutive eligible patients; no stratification or matching.	5
7	Precisely define all clinical endpoints examined.<br><br>Reported: Section 2.3: OS from surgery to death from any cause or last follow-up; DFS from surgery to first recurrence or death.	6
8	List all candidate variables initially examined or considered for inclusion in models.<br><br>Reported: Section 2.3: age, sex, emergency presentation, tumor site, neoadjuvant therapy, adjuvant chemotherapy, lymphovascular invasion, with stage and ASA class as strata.	6
9	Give rationale for sample size; if the study was designed to detect a specified effect size, give the target power and effect size.<br><br>Reported: Section 2.3: no formal sample size calculation; all consecutive eligible patients in the study period.	6
Statistical analysis methods		
10	Specify all statistical methods, including details of any variable selection procedures and other model-building issues, how model assumptions were verified, and how missing data were handled.<br><br>Reported: Section 2.3: Kaplan–Meier and log-rank; univariable and multivariable Cox models with prespecified covariates (no stepwise selection); separate models for collinear composite scores.	6
11	Clarify how marker values were handled in the analyses; if relevant, describe methods used for cutpoint determination.<br><br>Reported: Section 2.3: PNI analyzed as continuous (primary); dichotomized at the cohort median for interpretation	5, 15

	only; published cut-offs applied post hoc (Section 3.7, Table 7).	
**RESULTS**		
Data		
12	Describe the flow of patients through the study, including the number of patients included in each stage of the analysis (a diagram may be helpful) and reasons for dropout. Specifically, both overall and for each subgroup extensively examined report the numbers of patients and the number of events.<br><br>Reported: Section 3.1: 418 screened, 102 excluded, 316 analyzed; 312 in multivariable models.	7
13	Report distributions of basic demographic characteristics (at least age and sex), standard (disease-specific) prognostic variables, and tumor marker, including numbers of missing values.<br><br>Reported: Table 1 (age, sex, stage, ASA class, urgency, site, treatment, and index distributions) and Table 2.	7, 11
Analysis and presentation		
14	Show the relation of the marker to standard prognostic variables.<br><br>Reported: Section 3.2 and Table 2: PNI across stage, ASA class, presentation, tumor site, lymphovascular invasion, neoadjuvant and adjuvant therapy, and sex.	9–11
15	Present univariable analyses showing the relation between the marker and outcome, with the estimated effect (e.g., hazard ratio and survival probability). Preferably provide similar analyses for all other variables being analyzed. For the effect of a tumor marker on a time-to-event outcome, a Kaplan-Meier plot is recommended.<br><br>Reported: Section 3.3 and Table 3: univariable hazard ratios with 95% CIs for OS and DFS.	11
16	For key multivariable analyses, report estimated effects (e.g., hazard ratio) with confidence intervals for the marker and, at least for the final model, all other variables in the model.<br><br>Reported: Section 3.4 and Table 4: adjusted hazard ratios with 95% CIs for all model variables.	12
17	Among reported results, provide estimated effects with confidence intervals from an analysis in which the marker and standard prognostic variables are included, regardless of their statistical significance.<br><br>Reported: Section 3.4 and Table 4: PNI adjusted for standard prognostic variables, stratified by stage and ASA class.	12
18	If done, report results of further investigations, such as checking assumptions, sensitivity analyses, and internal validation.<br><br>Reported: Section 3.6 and Table 6: scaled Schoenfeld residuals, 36-month time-split model, upfront-surgery sensitivity analysis, Harrell C-index with bootstrap confidence intervals and likelihood-ratio testing.	14–15
**DISCUSSION**		
19	Interpret the results in the context of the pre-specified hypotheses and other relevant studies; include a discussion of limitations of the study.<br><br>Reported: Section 4 and Sections 4.1–4.4: interpretation against the stated objectives, with comparison to Turkish, Jordanian and Iranian cohorts and to a 43-study meta-analysis.	16–19
20	Discuss implications for future research and clinical value.<br><br>Reported: Sections 4.2, 4.3 and 5: clinical use for early risk stratification and nutritional optimization; need for locally derived thresholds and prospective multicenter validation.	17–19
